# The First Chinese Edited Babies: A Leap of Faith in Science

**DOI:** 10.5935/1518-0557.20190042

**Published:** 2019

**Authors:** Vera Lucia Raposo

**Affiliations:** 1 Faculty of Law, University of Macao, Macao, China

China is one of the leaders in the genetics game. Since February 2014, when Chinese scientists published their results on the use of the CRISPR-Cas9 technique in one-cell-stage monkey embryos (Niu *et al*., 2014), they have been the main drivers of this new technique.

In November 2018, media from all over the world reported that two twin girls had been born with modified genes to make them HIV immune. Their birth was the result of an ‘experiment' (presently it can only be called that) conducted by He Jiankui with couples in which the males were HIV carriers. Using CRISPR technology to immunise the babies against the HIV virus, He Jiankui managed to disable the CCR5 gene that enables the HIV infection (although he still did not present complete evidence of this achievement). However, Chinese existing regulation, thought not very detailed, does not provide legal basis for the experiment carried out by He Jiankui and his team (Nie, 2018; Nie & Cheung, 2019). In particular, the 2003 “Ethical Guiding Principles for Research on Embryonic Stem Cell” issued by China's Ministry of Science and Technology and then Ministry of Health (now National Health Commission), very clearly bans research to be performed on human *in vitro* embryos after the 14^th^ day of existence, and its subsequent implantation into a human uterus. Furthermore, in spite of the alleged reason for the genetic intervention related with the prevention of HIV, the scientific community also knows that the CCR5 gene is related with major brain functions. He Jiankui might have done some kind of human enhancement by created two especially intelligent human beings, with better memory and higher IQ (Joy *et al*., 2016).

This event has subsequently fuelled debate over CRISPR-Cas9, the most recent gene editing technique.

Genetic engineering has been around from some time. Pretty much every argument for and against it has already been presented, and national and international regulations tried to provide a legal and ethical answer to it, even if dubious and incomplete. Nonetheless, CRISPR-Cas9 has changed the way genetic engineering is done and this revolution might transform the entire perception on gene editing.

CRISPR-Cas9 is much simpler, cheaper and more precise than the previous methods of handling genes (Gyngell *et al*., 2017). On the other hand, while the previous methods have only allowed new elements to be added to the human genome, CRISPR-Cas9 has made it possible to add, delete or replace genes, thereby opening the door to new types of genetic interventions. One of its most promising achievements might be the possibility of reversing the effects of faulty procedures, to deal with eventual errors.

The technical improvements reached by CRISPR-Cas9 are far from irrelevant. The higher the level of precision and efficiency of CRISPR-Cas9, the greater the change in the general perception of genetic modification. Thus, in the future CRISPR-Cas9 might be considered as any other medical procedure.

Up until now, however, the objections raised against it have been multiple and diverse. Some are associated to the technical aspects of the procedure. Despite its increased precision, safety has continued to be a pressing concern. The risk of unexpected and undesired changes to a gene that is able to carry unpredictable consequences cannot be controlled. For instance, interventions with CCR5 genes, as in the case of the Chinese twins, carry a higher risk of infection from the West Nile virus and severe flu (Glass *et al*., 2006). Additionally, according to the scientists that analysed Jiankui's materials, gene editing was incomplete in at least one of the babies. Therefore, some issues for the children's future health may resulted from the outcomes of this procedure. Alternatively, even when the procedure is successful, it can only handle genetic disorders caused by a single gene and the fact is that most of the existing disorders are multigenetic. Nonetheless, one cannot rule out that further development of this technique will enable it to deal with several genes, even thousands of genes, at the same time. More importantly, it is expected that further research will make the procedure much more reliable, efficient and, therefore, safe.

The Chinese episode has also generated other issues. Several notes demonstrate that this was an experiment and not a therapeutic intervention (even He Jiankui called it a 'clinical trial'). The babies were not at risk of being born with HIV, given that sperm washing had been used so that only non-infected genetic material was used. Further, even though one of the parents (or both) was infected, it did not mean the children were more prone to becoming infected. The risk of becoming infected by the parents' virus was very low (Cowgill *et al*., 2008). In sum, there was no curative purpose, nor even the intention to prevent a pressing risk. Finally, the interventions were different for each twin. In one case, the two copies of CCR5 were modified, whereas in the other only one copy was modified. This meant that one twin could still become infected, although the evolution of the disease would probably be slower. The purpose of the scientific team was apparently to monitor the evolution of both babies and the differences in how they reacted to their different genetic modifications. This note also raised the issue of parents' informed consent regarding human experimentation, which follows a much stricter regimen than consent for therapeutic procedures.

Moreover, if indeed the genetic intervention in place enhanced the twins this opens the door to an all new discussion: can we use gene editing to create "better" (whatever that may be...) human beings, maybe even a super race of humans? The scenario, when presented like that, seems terrifying, but actually the story of mankind is nothing more than one of enhancement, so probably in the future we won't look at human amelioration with such suspicion (Raposo, 2019).

Ethical concerns have long been asserted against genetic interventions. However, most of the objections have been based more on prejudice than substantive arguments. Critics have invoked the sanctity of the human genome, as if changing it would equate to playing God (Habermas, 2003). However, protecting the human genome should not prevent genetic interventions that can improve our lives. What brings real value to our lives is having a genetic code that allows us to live free of severe diseases, not to have an unmodified but unhealthy genetic code. Some have argued the perils of genetic discrimination (Mehlman & Botkin, 1998) and eugenics (Habermas, 2003), but if that were truth no medical treatment would be allowed under the suspicion of discriminating the ones not that are not treated and of aspiring to create a “superior” society of healthy people. The risk of undermining the human genetic pool (Committee on Science, Technology, and Law, 2016) is also a recurrent concern, but “there are more than six billion humans on the planet. Absent some kind of magic wand, it is initially difficult to see how any given genetic intervention could change human nature” (McConnell, 2010). The eventual loss of our human nature (Habermas, 2003) has been also invoked, but changing our genes does not change our human nature. Humanity does not reside in a specific genetic code, but in a certain perception of the world and our role in it. That role adds to the story of how we overcome the surrounding environment and ourselves.

Until now, the scientific community has been quite critical of this procedure. What is in place right now is a precautionary principle (Committee on Science, Technology, and Law, 2016). Research has not been completely banned. It has been allowed when its aim has been to obtain additional data on the procedure's safety. Likewise, somatic gene editing (that is, genetic interventions that will not pass to offspring) has been allowed in humans, and germinal gene editing (genetic interventions that will be transmitted to progeny) has been allowed in non-humans. In sum, there has been a restriction on the kind of research permitted, and for the latter the requisites have been quite demanding (Guttinger, 2018). Most likely, it could not have been any other way. If not for the restrictions, the chances are that CRISPR-Cas9 would have been totally banned. Accordingly, the precautionary principle has been the alternative to absolute prohibition (Ellis, 2006; Guttinger, 2018).

I believe it is still too early to perform germinal gene editing (even resorting to CRISPR-Cas9) as a regular therapeutic procedure (Thrasher *et al*., 2016; Committee on Science, Technology, and Law, 2016; Friedmann *et al*., 2015; Kang *et al*., 2016). We still need to decode the amazing mysteries of genomics to understand how to safely use this procedure in human beings. The problem with the Chinese episode is not so much the use of gene editing, but its untimely use, without scientific evidence supporting the safety of CRISPR-Cas9. According with analysis done to Jiankui's work, “neither Lulu nor Nana possessed the 32-base pair deletion desired in the CCR5 gene, and each embryo instead expressed variants of various lengths. These novel mutations have not been previously shown to prevent HIV infection and may even be harmful. Some of He's data also suggest the presence of both edited and unedited cells, leading to a phenomenon called mosaicism, as well as off-target effects of the edit that could cause other unanticipated changes in the genome” (Nie & Cheung, 2019).

It is a fact that the technique has already been used in somatic therapeutic interventions with success, reaching goals that regular medical treatments cannot achieve (Cyranoski, 2016). Nonetheless, somatic interventions and the risk of passing genetic modifications, including genetic errors, to offspring raise several technical and ethical issues that must be addressed.

The Chinese experience was even more daunting, because in addition to being a somatic intervention it was not ‘necessary' to the embryos' well-being, i.e., the embryos were healthy and the experiment merely performed a health enhancement and eventually also a non-health related enhancement.

In the future, when they are properly developed, CRISPR-Cas9 and gene editing in general can become very useful tools to deal with health-related issues. This not only includes purely therapeutic (curative) interventions, but also health-related enhancements, such as immunising a person against certain viruses (similarly to what currently happens with vaccines), just like in the Chinese experiment. If or when CRISPR-Cas9 is properly developed, it can be used as a regular medical treatment (in broad terms, including preventive measures).

Therefore, we cannot impose a ban on research in this domain, even in spite of this episode. If scientific accuracy is the goal, and indeed, it is, this goal can only be achieved by investing in more research. I do understand that some caution is required, not only to prevent genetic mistakes that we may be unable to undo, but also to allow enough time to find better answers to the legal and ethical dilemmas involved. Nonetheless, we need to continue. Stopping here would mean to waste decades of investigation and lose a brilliant opportunity to provide greater well-being for humankind. People now and in the future could be spared the pain and suffering caused by the many diseases for which we still do not have a cure. The answer may well be in the genes.
